# Peroral endoscopic tunneling under saline combined with partial myotomy for hypercontractile esophagus

**DOI:** 10.1055/a-2563-1534

**Published:** 2025-03-28

**Authors:** Georgios Mavrogenis, Alexandros Ioannou, Thanassis Karamountzos, George Karamanolis

**Affiliations:** 1 Department of Gastroenterology, Unit of Hybrid Interventional Endoscopy, Mediterraneo Hospital, Athens, Greece; 2 Department of Gastroenterology, Alexandra General Hospital, Athens, Greece; 3 Department of Gastroenterology and Hepatology, Laiko General Hospital, National and Kapodistrian University of Athens, Athens, Greece

Underwater peroral endoscopic myotomy was initially presented as an alternative approach for the treatment of achalasia, with only a few case reports published since its initial description 1 2 3 4 . The theoretical advantage of using saline infusion instead of carbon dioxide is the diminished risk of gas-related events such as capnoperitoneum, tension pneumothorax, or pneumomediastinum. However, in our experience, the major advantage of working under saline is the stabilization of the endoscope in the setting of increased esophageal motility. The purpose of this video ( Video 1 ) is to illustrate the advantages of performing peroral endoscopic tunneling under saline combined with partial myotomy in the setting of hypercontractile esophagus.

Demonstration of peroral endoscopic tunneling under saline combined with partial myotomy for hypercontractile esophagus.Video 1

In this rare disorder, the increased motility of the esophagus ( Fig. 1 ) makes the procedure challenging and raises the risk of inadvertent mucosal damage. However, by performing the dissection under saline ( Fig. 2 ), the mucosa floats away from the muscle layer and the spasms of the esophagus do not interfere with the dissection plane. In addition, by performing partial myotomy ( Fig. 3 ) during tunneling, the axis of the tunnel is straightened, and the intensity of contractions is significantly diminished. When both techniques are applied, the procedure becomes safer and faster. Once the tunnel is completed the saline is aspirated in order to diminish the risk of postoperative pleural effusions, and standard myotomy is performed ( Fig. 4 , Fig. 5 ).

**Fig. 1:**
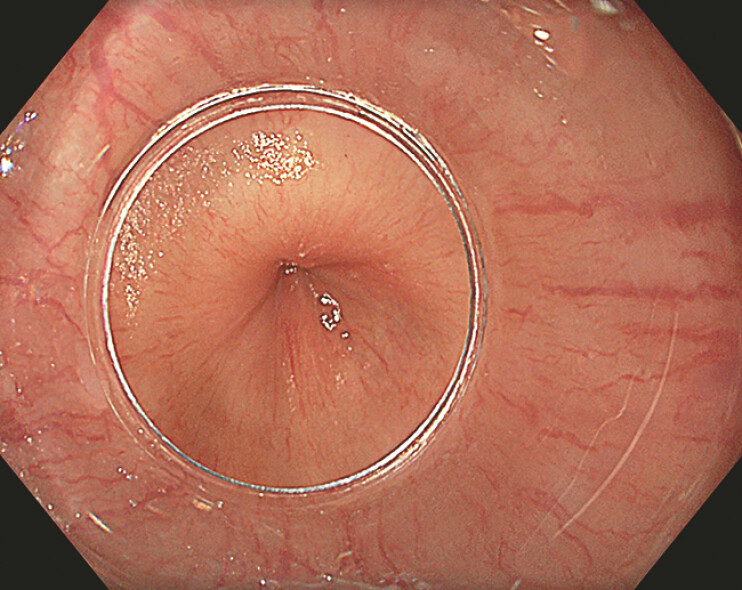
Intense esophageal contractions in a case of hypercontractile esophagus.

**Fig. 2:**
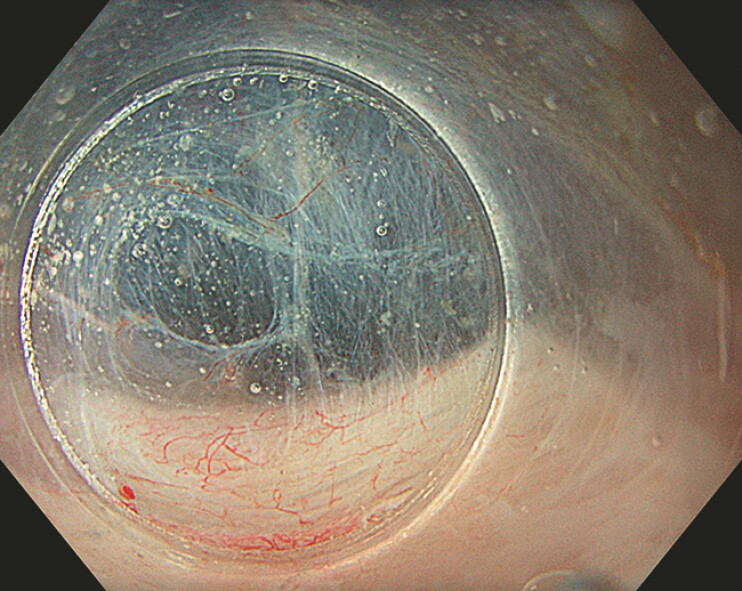
Tunneling under saline. The view is magnified, the mucosa floats away from the muscle layer, and the contractions do not interfere with the dissection plane.

**Fig. 3:**
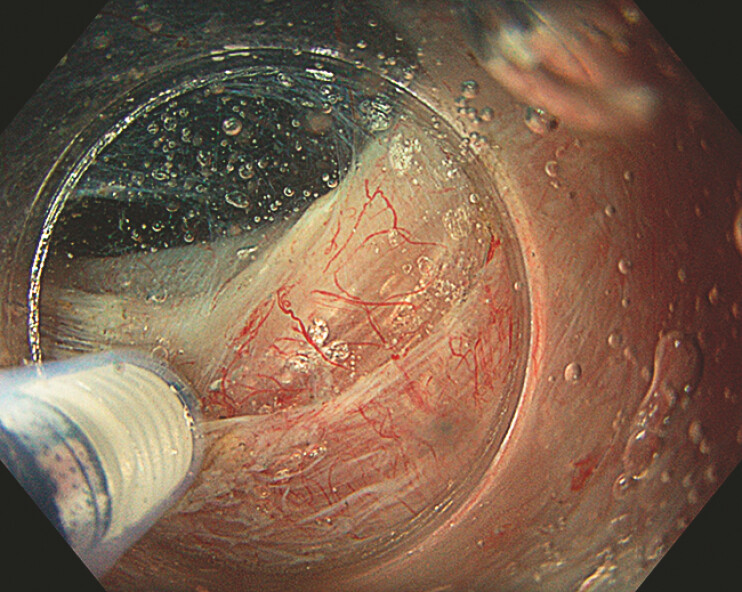
Partial myotomy performed under saline.

**Fig. 4:**
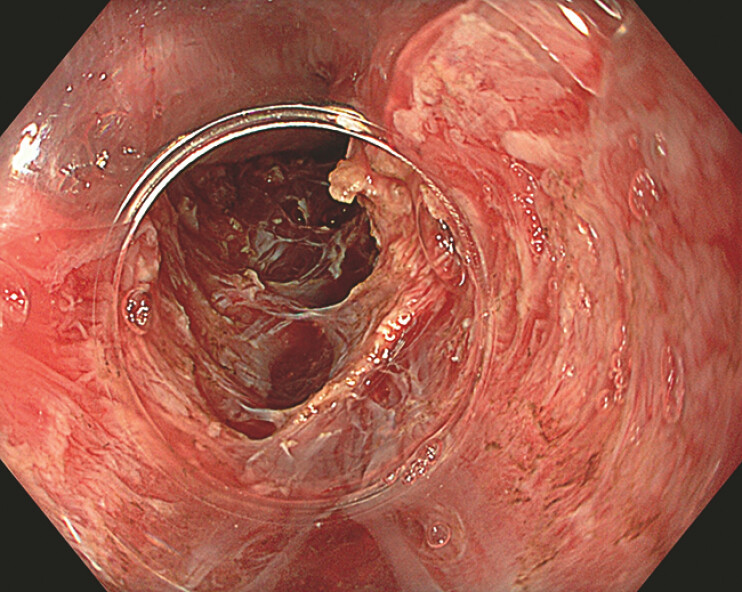
Full-thickness myotomy.

**Fig. 5:**
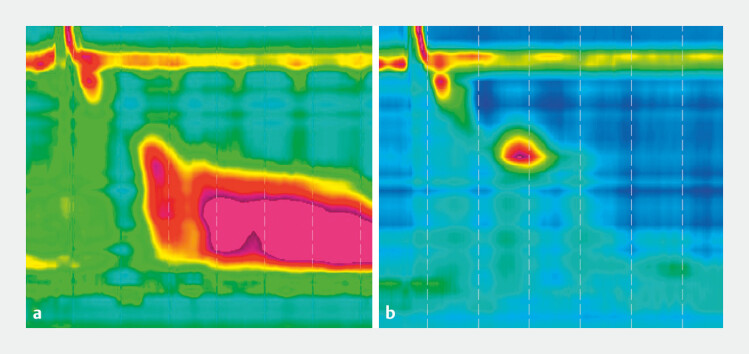
High-resolution manometry before ( **a** ) and after ( **b** ) myotomy, showing loss of hypercontractility.

In conclusion, we believe that tunneling under saline combined with partial myotomy is an innovative approach for faster and safer dissection in motility disorders with intense esophageal contractions.

Endoscopy_UCTN_Code_TTT_1AO_2AP

## Notes

Endoscopy E-Videos https://eref.thieme.de/e-videos
*E-Videos* is an open access online section of the journal *Endoscopy* , reporting on interesting cases and new techniques in gastroenterological endoscopy. All papers include a high-quality video and are published with a Creative Commons CC-BY license. Endoscopy E-Videos qualify for HINARI discounts and waivers and eligibility is automatically checked during the submission process. We grant 100% waivers to articles whose corresponding authors are based in Group A countries and 50% waivers to those who are based in Group B countries as classified by Research4Life (see: https://www.research4life.org/access/eligibility/ ). This section has its own submission website at https://mc.manuscriptcentral.com/e-videos .

## References

[1] Binmoeller KF Bhat YM Underwater peroral endoscopic myotomy Gastrointest Endosc 2016 83 454 10.1016/j.gie.2015.08.066 26358328

[2] Uchima H Colan J Marín I Underwater peroral endoscopic myotomy (u-POEM) after tension capnoperitoneum and capnothorax during POEM Endoscopy 2020 52 E396 E397 32303086 10.1055/a-1144-2547

[3] Sferrazza S Calabrese G Maselli R Underwater techniques in gastrointestinal endoscopy: diving into the depths Cancers (Basel) 2024 16 3535 39456629 10.3390/cancers16203535 PMC11506518

[4] Capogreco A de Sire R Massimi D Underwater coagulation using hybrid knife in peroral endoscopic myotomy for achalasia Endoscopy 2024 56 547 548 10.1055/a-2258-8371 38936349 PMC11211000

